# AI-assisted ascites volumetry for prognostic stratification in HCC treated with Atezolizumab/Bevacizumab

**DOI:** 10.1186/s40644-026-01105-w

**Published:** 2026-08-11

**Authors:** Maximilian Moos, Daniel Bender, Friedrich Foerster, Lucas Wiesmann, Arndt Weinmann, Roman Kloeckner, Constantin Scholz, André Euler, Aline Mähringer-Kunz, Tobias Bäuerle, Fabian Stoehr, Felix Hahn, Lukas Müller

**Affiliations:** 1 Department of Diagnostic and Interventional Radiology, University Medical Center of the Johannes Gutenberg University Mainz, Langenbeckstr. 1, 55131 Mainz, Germany; 2 Department of Internal Medicine I, University Medical Center of the Johannes Gutenberg University Mainz, Mainz, Germany; 3 Institute of Interventional Radiology, University Hospital Schleswig-Holstein, Lübeck, Germany; 4 Department of General, Visceral and Transplant Surgery, University Medical Center of the Johannes Gutenberg University Mainz, Mainz, Germany; 5 Department of Radiology, Kantonsspital Baden, Affiliated Hospital for Research and Teaching of the Faculty of Medicine of the University of Zurich, Im Ergel 1, Baden, 5404 Switzerland

**Keywords:** Hepatocellular carcinoma (HCC), Ascites volumetry, Artificial intelligence (AI), Atezolizumab/Bevacizumab, Prognostic imaging biomarkers

## Abstract

**Purpose:**

Portal hypertension-related parameters are known to have important prognostic implications in patients with hepatocellular carcinoma (HCC). Ascites has consistently been associated with impaired outcomes. However, the prognostic relevance of quantified ascites volume in patients undergoing Atezolizumab/Bevacizumab remains unclear.

**Methods:**

90 patients with HCC treated with Atezolizumab/Bevacizumab between 2020 and 2024 were retrospectively included. Ascites was assessed on baseline contrast-enhanced CT imaging using AI-based pre-segmentation followed by manual correction to quantify ascites volume. Overall survival (OS) was analyzed using Kaplan–Meier analysis and Cox regression. Ascites volume was analyzed both as a continuous variable and using predefined volumetric cutoffs (< 613.5 mL and ≥ 613.5 mL).

**Results:**

Ascites was present in 39% of patients, with a median ascites volume of 344 mL (IQR 29–929). When analyzed as a continuous variable, ascites volume was significantly associated with impaired OS in univariate analysis (Hazard Ratio (HR) 2.88, *p* < 0.001) but not after multivariate adjustment (*p* = 0.214). Stratification by ascites volume revealed a stepwise reduction in OS, with patients without ascites showing the best survival, followed by patients with low-volume ascites (< 613.5 ml), and patients with high-volume ascites (≥ 613.5 ml) demonstrating the poorest outcomes (log-rank *p* < 0.001). In multivariate analysis, high-volume ascites remained independently associated with impaired OS (HR 4.01, *p* < 0.001), whereas low-volume ascites did not.

**Conclusion:**

Ascites volume showed a threshold-dependent prognostic effect in patients with HCC treated with Atezolizumab/Bevacizumab. Volumetric staging identifies high-volume ascites as an independent marker of poor outcome and may provide more clinically relevant risk stratification than binary ascites assessment.

**Clinical trial number:**

Not applicable.

## Introduction

Hepatocellular carcinoma (HCC) represents one of the most prevalent malignant diseases worldwide. According to the National Cancer Institute, primary liver cancer, of which HCC accounts for the majority of cases, ranks among the most common malignancies globally and remains a leading cause of cancer-related mortality, particularly in men [1, 2]. For staging, treatment allocation, and prognostic assessment, current guidelines of the European Association for the Study of the Liver (EASL) and the American Association for the Study of Liver Diseases (AASLD) recommend the Barcelona Clinic Liver Cancer (BCLC) classification system [3, 4].

For a long time, therapeutic options for patients with advanced-stage hepatocellular carcinoma (BCLC stage C) were limited, with Sorafenib representing the only recommended systemic treatment [5]. The therapeutic landscape has shifted fundamentally following the results of the IMbrave150 trial, which established the combination of Atezolizumab (anti–PD-L1 antibody) and Bevacizumab (anti-VEGF antibody) for patients with unresectable or metastatic hepatocellular carcinoma (BCLC stage C) [6]. Importantly, the application of Atezolizumab/Bevacizumab is also being explored beyond advanced disease, such as in selected patients with intermediate-stage HCC (BCLC stage B), within the framework of stage migration concepts [7–9].

Most HCC develop in patients with underlying liver cirrhosis, which represents the most important risk factor for HCC [3]. Progressive cirrhosis leads to characteristic alterations in splanchnic hemodynamics and portal hypertension, frequently accompanied by sodium and water retention [10]. These pathophysiological changes culminate in the development of ascites, one of the most common manifestations of hepatic decompensation [11]. Parameters associated with portal hypertension have repeatedly been shown to carry important prognostic implications in patients with HCC [12–14].

Ascites has consistently been identified as an adverse prognostic factor in patients with hepatocellular carcinoma undergoing both systemic and locoregional therapies [15, 16]. Beyond its mere presence, several studies have demonstrated that the extent of ascites further stratifies prognosis, particularly in patients undergoing liver resection or TACE [12, 17].

Therefore, the aim of this study was to investigate the prognostic significance of quantified ascites volume in HCC patients undergoing systemic therapy with Atezolizumab/Bevacizumab. Our hypothesis was that not only the presence of ascites but particularly its precise volume has a decisive impact on the prognosis of this patient group.

## Methods

### Study designs and ethics

The Ethics Committee of the Medical Association of Rhineland Palatinate, Mainz, Germany approved this study and waived requirement for informed consent for the retrospective analysis of clinical data (permit number 2021–15,984). This study was conducted following the ethical principles of the Declaration of Helsinki. Patient records and information were anonymized prior to analysis.

### Patients

Between 01.01.2020 and 01.05.2024, 90 patients fulfilled the inclusion criteria: (1) age above 18 years; (2) histologically or image-derived HCC diagnosis based on the EASL criteria; (3) no liver transplantation or tumor resection during the follow-up period; (4) treatment with Atezolizumab/Bevacizumab (5) baseline contrast-enhanced CT scan for ascites volume assessment; (6) full availability of clinical, laboratory, and imaging data.

### Data acquisition

All patient data were retrospectively retrieved from our institution’s clinical and radiological information systems (Mesalvo, Freiburg, Germany) and the local picture archiving and communication system (Sectra, Linköping, Sweden). Additional laboratory data were acquired from the laboratory database (Lauris, Donaueschingen, Germany). The final dataset included all available data on patient demographics, clinical assessments of the underlying liver disease and tumor, imaging and laboratory parameters.

### Ascites volume assessment

In a first step, a 3D U-Net for ascites segmentation was set up using the MONAI framework [18]. A model with five encoding levels (16, 32, 64, 128, and 256 channels) was trained using the Tversky loss and the Adam optimizer. The training cohort was based on 102 therapy-naive patients with HCC and ascites prior to first TACE for whom segmentations were available from a previous study [12].

Model performance was evaluated in an a priori defined holdout validation cohort comprising 24 patients undergoing alternative immunotherapies (Pembrolizumab *n* = 6, Nivolumab *n* = 6, Atezolizumab/Bevacizumab *n* = 12) [19].

Within the study cohort comprising 90 patients treated with Atezolizumab/Bevacizumab, the presence of ascites was evaluated in the Picture Archiving and Communication System (PACS; Sectra, Linköping, Sweden). For patients with ascites, the portal venous phase of the abdominal CT scans was extracted and pre-segmentations computed by the U-Net.

**Fig. 1:**
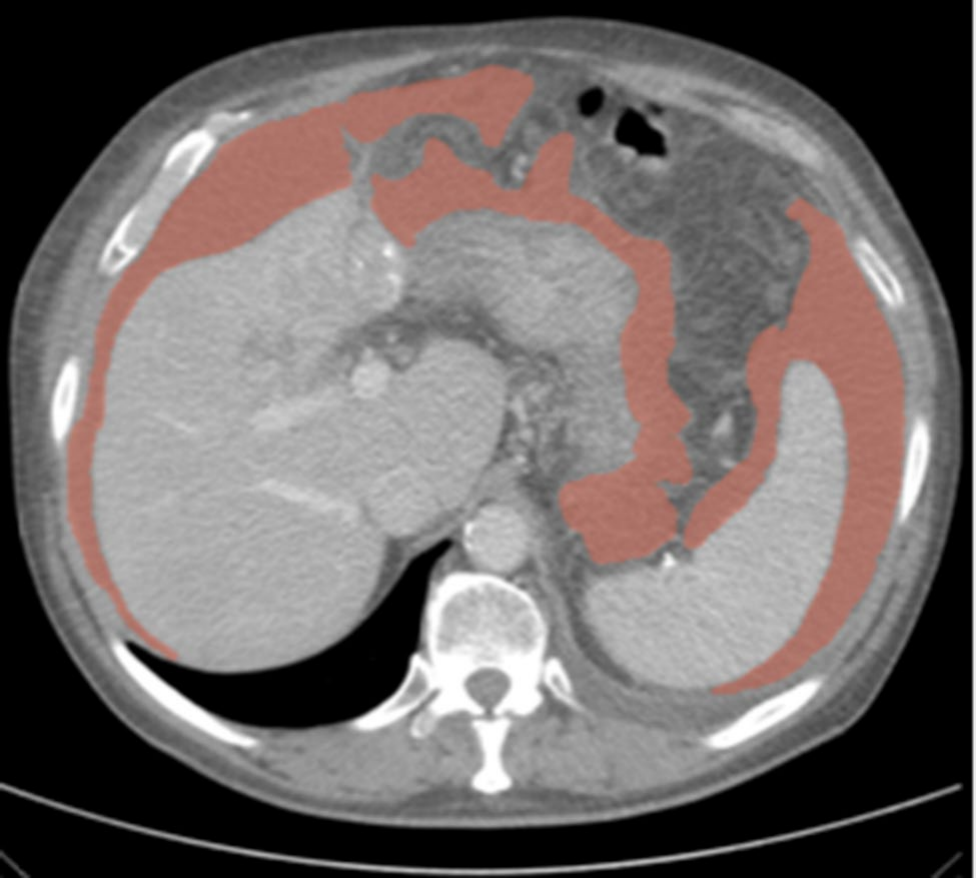
Example of AI-based segmentation (red) of ascites on axial abdominal CT

Finally, the AI-based pre-segmentations were reviewed and corrected by the same board-certified radiologist and approved using 3D Slicer (Fig. 1) [20]. Using a single reviewer ensured methodological consistency across all volumetric assessments; however, intra- and interreader reproducibility of segmentation corrections were not formally assessed in the current study.

### Statistical analysis

All statistical analyses and graphics were performed using R Studio (RStudio Team [2020]. RStudio: Integrated Development for R. RStudio, PBC, http://www.rstudio.com, last accessed November 20, 2025) and R 4.0.3 (A Language and Environment for Statistical Computing, R Foundation for Statistical Computing, http://www.R-project.org). Continuous data were reported as mean ± standard deviation, while categorical and binary parameters were reported as absolute numbers and percentages.

Overall survival was analyzed using Cox proportional hazards regression models. Survival data including dates of death were obtained from the institutional hospital information system (HIS). Univariate Cox regression analyses were first performed to evaluate the association between each parameter of interest and survival. Variables with clinical relevance and/or statistical indication were subsequently entered into multivariate Cox regression models to adjust for potential confounding factors.

Ascites volume was initially analyzed as a continuous variable in separate univariate and multivariate models. In addition, predefined ascites volume cutoffs, derived from the study by Müller et al. (< 613.5 ml and ≥ 613.5 ml) [12] were assessed in independent univariate and multivariate Cox regression models. Furthermore, the optimal ascites threshold for stratification in our cohort was determined using an outcome-oriented cut-off selection method, commonly referred to as optimal stratification, using the surv_cutpoint function (https://www.rdocumentation.org/packages/survminer/versions/0.5.2/topics/surv_cutpoint) in R. This approach identifies the value of a continuous variable that best separates patients into prognostically distinct groups with respect to survival.

A p-value < 0.05 was considered statistically significant.

## Results

### AI ascites segmentation

**Fig. 2:**
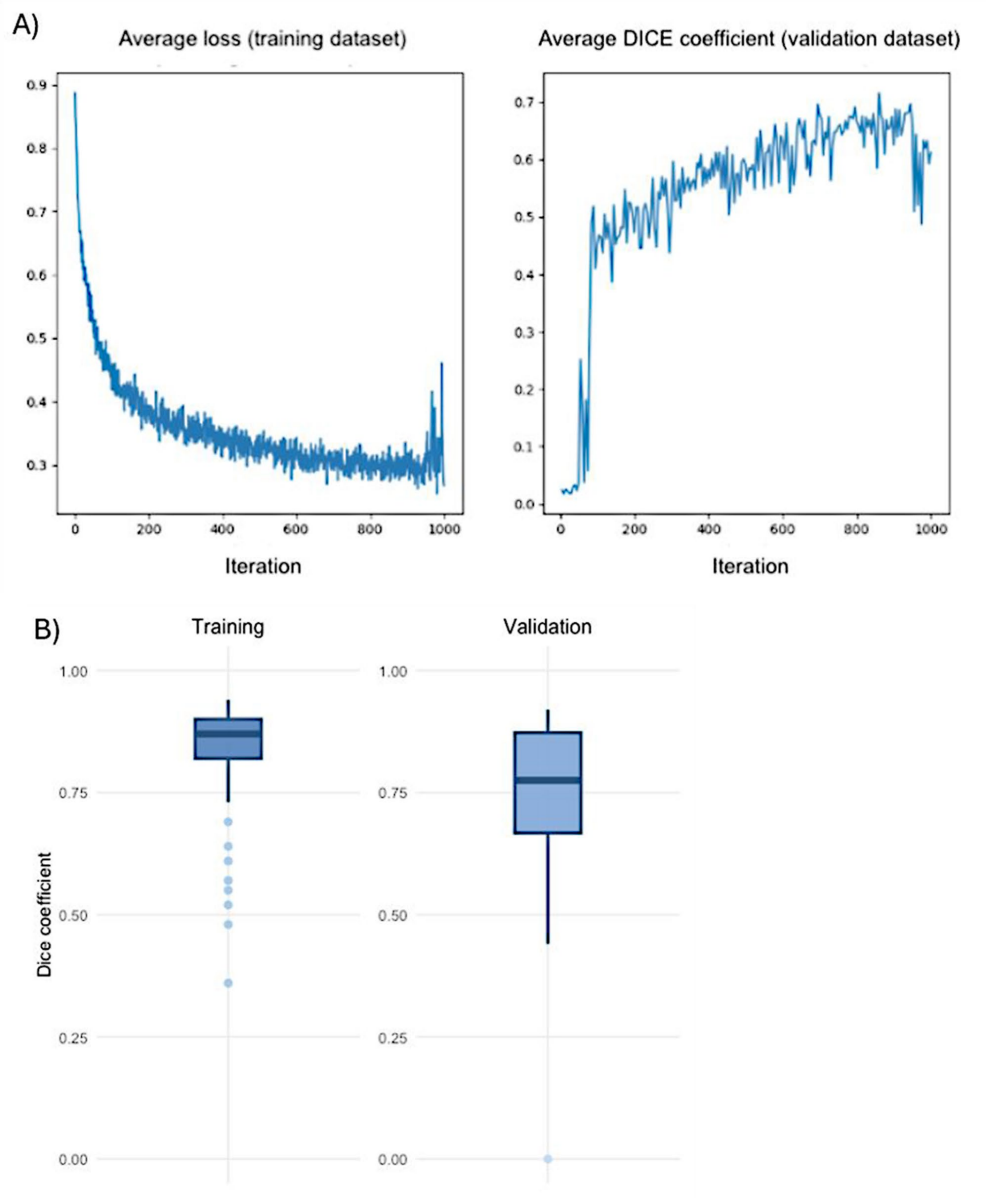
**(A)** Progression of the loss function on the training dataset and of the DICE coefficient on the validation dataset during training. (**B**) Boxplot of Case-wise Dice Scores on the training and validation

Training showed a steady decrease in training loss and a concomitant increase in the validation DICE coefficient, indicating progressive performance gains. Temporary declines in validation performance were consistent with overfitting effects during later epochs. The final model achieved an average DICE coefficient of 0.84 (median 0.87) on the training set and 0.72 (median 0.775) on the holdout validation cohort, demonstrating moderate segmentation accuracy and generalizability (Fig. 2).

### Baseline characteristics

A total of 90 patients with HCC treated with Atezolizumab/Bevacizumab were included in the analysis. The median age was 70 years (IQR 62–76), and the majority were male (79%). Ascites was present in 35 patients (39%), with a median ascites volume of 344 mL (IQR 29–929). Previous therapy regimes, if any and further baseline characteristics are detailed in Table 1.

**Table 1:** Baseline characteristics of the study population

Variable	All Patients (*n* = 90)
Age	70 (62–76)
Sex, n (%)	
Female	19 (21%)
Male	71 (79%)
Leading etiology of liver disease, n (%)	
Alcohol	28 (31%)
AIH	1 (1%)
Cryptogenic	3 (3%)
HBV	11 (12%)
HCV	16 (18%)
Hemochromatosis	2 (2%)
MASH	17 (19%)
PBC	1 (1%)
no liver disease	11 (12%)
ALBI Grade, n (%)	
1	9 (10%)
2	66 (73%)
3	15 (17%)
Ascites	
Presence of Ascites, n (%)	35 (39%)
Volume in cases of Ascites, mL, median (IQR)	344 (29–929)
BCLC Stage, n (%)	
A	2 (2%)
B	20 (22%)
C	67 (75%)
D	1 (1%)
Child Pugh Stage, n (%)	
A	63 (70%)
B	17 (19%)
C	10 (11%)
ECOG, n (%)	
0	27 (30%)
1	46 (51%)
2	17 (19%)
AFP, ng/ml, median (IQR)	115 (7-2226)
Albumin, g/l, median (IQR)	32,0 (29.0–36.0)
Bilirubin, mg/dl, median (IQR)	1,20 (0.70–1.80)
INR, median (IQR)	1,20 (1.10–1.40)
Creatinine, median (IQR)	0,80 (0,68 − 1,02)
Previous Therapy	
TACE	28 (31,1%)
TACE + systemic therapy	5 (5,6%)
Resection	15 (16,7%)
Resection + systemic therapy	2 (2,2%)
Thermal ablation	2 (2,2%)
Systemic therapy	2 (2,2%)
No previous therapy	36 (40%)
Time follow-up, days. median (IQR)	269 (133–539)

### Ascites volume, univariate and multivariate analysis

In a first step, ascites volume was analyzed as a continuous variable to assess its association with overall survival. In univariate Cox regression analysis, increasing ascites volume was significantly associated with impaired overall survival (Hazard Ratio (HR) 2.88, 95% CI 1.78–4.67, p-value < 0.001) (Table 2).

After adjustment for ECOG performance status, albumin, bilirubin, and creatinine in multivariate Cox regression analysis, ascites volume no longer remained independently associated with overall survival (HR 1.45, 95% CI 0.81–2.62, p-value 0.214), whereas albumin, bilirubin, and creatinine retained prognostic relevance (Table 2).

**Table 2:** Univariate and multivariate Cox proportional hazards regression analysis of baseline clinical and laboratory parameters and ascites at baseline

	Univariate			Multivariate		
Covariate	HR	95% CI	*P*-value	HR	95% CI	*P*-value
Gender (male)	1.81	0.95–3.47	0.073	-	-	-
Ascites	2.88	1.78–4.67	< 0.001	1.45	0.81–2.62	0.214
ECOG	1.60	1.10–2.34	0.014	0.99	0.64–1.54	0.981
BCLC stage A	reference					
BCLC stage B	0.32	0.07–1.42	0.135	-	-	-
BCLC stage C	0.29	0.07–1.20	0.087	-	-	-
BCLC stage D	0.46	0.04–5.08	0.525	-	-	-
Child-Pugh A	reference					
Child-Pugh B	1.76	0.95–3.24	0.070	-	-	-
Child-Pugh C	1.77	0.86–3.65	0.113	-	-	-
AFP	1.00	1.00–1.00	0.096	-	-	-
Albumin	0.86	0.82–0.91	< 0.001	0.90	0.85–0.96	< 0.001
Bilirubin	1.66	1.37–2.02	< 0.001	1.33	1.04–1.69	0.024
INR	1.05	0.62–1.79	0.848	-	-	-
Creatinine	2.36	1.10–5.07	0.027	2.84	1.26–6.41	0.012
Platelets	0.998	0.99-1.00	0.234	-	-	-

Hazard ratios (HRs) are reported with 95% confidence intervals (CI). Continuous variables were analyzed per unit increase. BCLC stage A and Child–Pugh class A served as reference categories. Variables included in the multivariate model were selected based on clinical relevance and univariate association. Ascites was analyzed as a continuous variable

In a second step, the study population was stratified into three groups based on ascites volume: patients without ascites (*n* = 55), patients with ascites volume < 613.5 ml (*n* = 21) and patients with ascites volume ≥ 613.5 ml (*n* = 14).

**Fig. 3:**
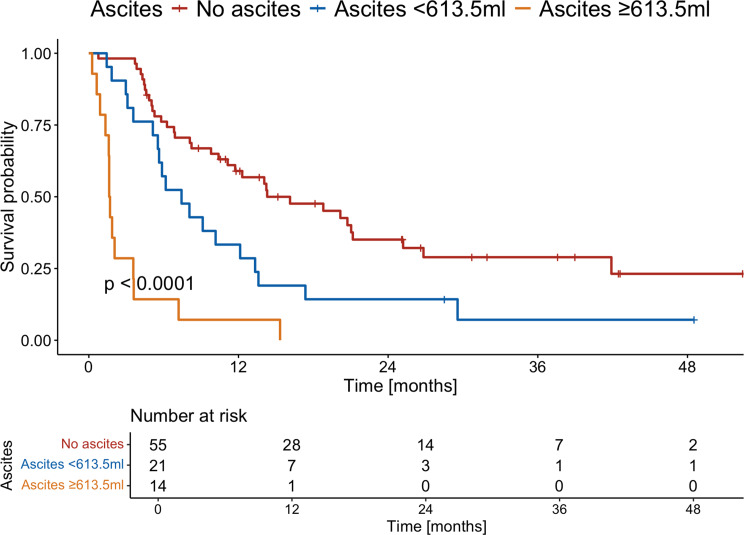
Kaplan–Meier analysis of overall survival stratified by ascites volume. Patients were categorized into three groups: no ascites, ascites volume < 613.5 ml, and ascites volume ≥ 613.5 ml. Survival differences between groups were assessed using the log-rank test

Kaplan–Meier survival analysis demonstrated a stepwise reduction in overall survival with increasing ascites volume (Fig. 3). Patients without ascites showed the best survival, followed by patients with an ascites volume < 613.5 ml, whereas patients with an ascites volume ≥ 613.5 ml showed impaired survival. The difference between groups was statistically significant (log-rank *p* < 0.001).

In univariate Cox regression analysis using the stratified ascites volume categories, patients with an ascites volume < 613.5 ml had a significantly increased risk of death compared with patients without ascites (HR 2.00, 95% CI 1.10–3.50; *p* = 0.015). Patients with an ascites volume ≥ 613.5 ml demonstrated a substantially higher risk of death (HR 8.40, 95% CI 4.30–16.40; *p* < 0.001) (Table 3).

After adjustment for ECOG performance status, albumin, bilirubin, and creatinine, an ascites volumes ≥ 613.5 ml remained independently associated with impaired overall survival (HR 4.01, 95% CI 1.82–8.84; *p* < 0.001). In contrast, an ascites volume < 613.5 ml was no longer independently associated with survival compared with patients without ascites (HR 1.00, 95% CI 0.51–1.97; *p* = 0.999) (Table 3).

**Table 3:** Univariate and multivariate Cox proportional hazards regression analysis of ascites volume categories and baseline clinical and laboratory parameters

		Univariate			Multivariate		
Covariate	*n*	HR	95% CI	*P*-value	HR	95% CI	*P*-value
No Ascites	55	reference					
Ascites < 613.5	21	2.0	1.1–3.5	0.015	1.00	0.51–1.97	0.999
Ascites ≥ 613.5	14	8.4	4.3–16.4	< 0.001	4.01	1.82–8.84	< 0.001
ECOG	90	**1.6**	**1.10–2.34**	**0.014**	0.91	0.59–1.41	0.675
Albumin	90	**0.86**	**0.82–0.91**	**< 0.001**	0.91	0.85–0.97	< 0.004
Bilirubin	90	**1.66**	**1.37–2.02**	**< 0.001**	1.41	1.09–1.81	0.009
Creatinine	90	**2.36**	**1.10–5.07**	**0.027**	2.12	0.89–5.01	0.089

HRs are shown with 95% CI. Patients without ascites served as the reference category. Continuous variables were analyzed per unit increase. Variables included in the multivariate model were selected based on clinical relevance and univariate analysis. Bold values indicate variables already included in the univariate analysis in Table 2

**Fig. 4:**
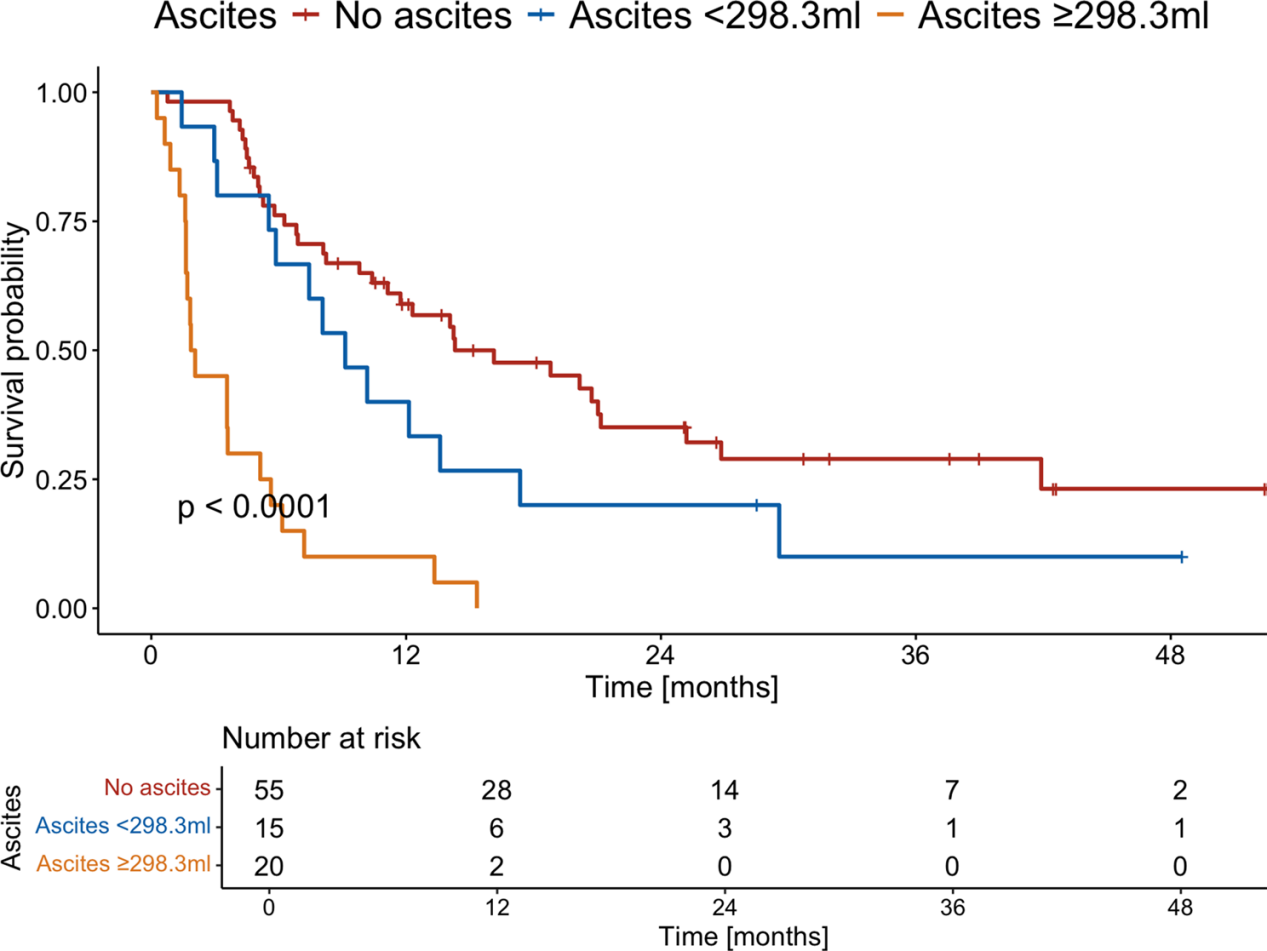
Kaplan–Meier analysis of overall survival stratified by the optimal ascites cutoff. Patients were categorized into two groups: ascites volume < 298.3 ml and ascites volume ≥ 298.3 ml. Survival differences between groups were assessed using the log-rank test

Additionally, a Kaplan–Meier survival analysis was performed using the optimal ascites cutoff determined for our cohort (Fig. 4). Patients were stratified into two groups based on an ascites volume of 298.3 ml. Patients with an ascites volume < 298.3 ml demonstrated improved overall survival compared to those with an ascites volume ≥ 298.3 ml. The difference between groups was statistically significant (log-rank *p* < 0.0001).

## Discussion

In the present study, we investigated the prognostic relevance of ascites volume in patients with HCC treated with Atezolizumab/Bevacizumab, with a focus on quantitative volumetric assessment. Our results demonstrate that ascites volume analyzed as a continuous variable was associated with a significant increased hazard ratio in univariate analysis. Moreover, stratification of patients according to ascites volume identified a subgroup of patients with high-volume ascites with poor survival where significance persisted in multivariate analysis.

The initial finding that ascites volume was univariately associated with survival when modeled as a continuous variable is in line with previous studies evaluating presence of ascites as a prognostic marker in HCC under systemic therapy with Sorafenib or Atezolizumab/Bevacizumab [21–23].

However, our subsequent stratified analysis revealed a different and clinically relevant perspective. By categorizing patients into no ascites, low-volume ascites (< 613.5 ml), and high-volume ascites (≥ 613.5 ml), ascites burden became independently associated with survival in patients with high-volume ascites, even after multivariate adjustment. The cutoff of 613.5 ml was derived from the study by Müller et al., who demonstrated that ascites volume quantified on CT imaging was an independent prognostic factor in patients undergoing TACE [12]. Importantly, our additional analysis using a cohort-specific optimal cutoff (298.3 ml) further supports the threshold-dependent prognostic effect of ascites. This cutoff is close to the 300 ml threshold reported by Wan et al., suggesting a consistent clinically relevant lower-volume boundary across studies [24]. Together, these findings indicate that multiple volumetric thresholds may better capture the prognostic impact of ascites.

Our findings extend this concept to patients receiving immunotherapy-based systemic treatment and support the notion that volumetric grading or staging of ascites, rather than a purely continuous assessment, better captures its threshold-dependent prognostic impact.

Of particular interest is the intermediate group of patients with low-volume ascites (< 613.5 ml). Based on Kaplan–Meier survival analysis, patients with low-volume ascites demonstrated worse survival than patients without ascites, but more favorable outcomes than those with high-volume ascites. This observation is consistent with previous studies in cirrhosis cohorts, which have shown that patients with sonographic mild (grade 1) ascites have a more favorable prognosis than those with moderate or severe (grade 2–3) ascites, while still exhibiting worse outcomes compared with patients without ascites [25–27]. These findings support the concept of low-volume ascites representing an intermediate prognostic stage. Future studies are warranted to investigate whether a more granular volumetric staging of ascites is clinically and therapeutically meaningful.

In patients with liver cirrhosis, ascites has long been categorized using sonographic and clinical staging systems reflecting increasing fluid burden. Traditionally, ascites is classified into three grades: grade 1, detectable only by ultrasound; grade 2, characterized by moderate, clinically apparent abdominal distension; and grade 3, representing large or tense ascites [28–30]. However, ultrasound-based assessment remains limited by operator dependency, patient habitus, and its inherently two-dimensional nature. CT-based volumetric quantification allows a comprehensive, three-dimensional, and reproducible assessment of ascites, capturing the entire peritoneal fluid compartment [31, 32]. While CT volumetry represents a logical next step in the evolution from qualitative sonographic staging toward precise, quantitative stratification of ascites burden with potential prognostic relevance, due to its time constraints, segmentation and volumetric assessment of ascites is not performed in clinical routine. AI tools can overcome this burden, however, given the complexity and highly irregular morphology of ascites with frequently ill-defined fluid boundaries, the achieved DICE coefficients were lower than those commonly reported for solid organ segmentations such as the liver or spleen [31]. Consequently, manual corrections were still associated with considerable time expenditure in the current study. Nevertheless, segmentation performance and thereby correction time may further improve with the availability of larger and more diverse annotated training datasets for ascites segmentation.

Importantly, for the clinically relevant distinction between low- and high-volume ascites, ultra-precise pixel-level segmentation may not be mandatory, as robust volumetric approximation may already be sufficient for clinically meaningful stratification. Therefore, even AI-based segmentations with moderate DICE performance could still provide substantial clinical utility. With further improvements in training data and model performance, fully automated volumetric assessment without extensive radiologist correction may become feasible, potentially enabling integration into routine clinical workflows.

It should also be noted that, beyond our chosen 3D U-Net architecture implemented within the MONAI framework, which has been established in our group over several years, more recent architectures such as nnU-Net have demonstrated highly promising segmentation performance across a variety of medical imaging tasks. Future studies may therefore benefit from evaluating these newer approaches, which could potentially further improve segmentation accuracy and reduce the need for manual correction.

Several limitations of this study should be acknowledged. First, the monocentric design and the limited sample size restrict the generalizability of our findings. This limitation is particularly relevant after stratification into ascites subgroups, resulting in relatively small numbers in the high-volume ascites group. In addition, the comparatively small cohort size in relation to the number of variables included in the multivariable analyses may have increased the risk of model overfitting and instability of effect estimates. Therefore, the identified associations should be interpreted cautiously and require validation in larger independent cohorts. Furthermore, ascites volume may partly reflect the severity of underlying portal hypertension and impaired liver function. Other manifestations of clinically significant portal hypertension, such as paraesophageal varices or prior variceal bleeding, as well as established liver function parameters (e.g., MELD score), were not systematically incorporated into the analyses and may therefore have acted as residual confounding factors. Moreover, ascites volume was assessed at a single baseline time point; dynamic changes in ascites burden during therapy were not evaluated and may provide additional prognostic information. Although the cutoff of 613.5 ml was derived from prior literature, the proposed volumetric threshold should be considered exploratory in the context of immunotherapy-treated patients. External validation in larger independent and preferably prospective multicenter immunotherapy cohorts is required before broad clinical application can be recommended. Finally, all AI-generated segmentations were reviewed and corrected by a single radiologist. Although this approach ensured consistency across cases, review of AI-generated segmentations may have introduced a certain degree of confirmation bias. Furthermore, potential effects of CT acquisition variability and segmentation inaccuracies on volumetric measurements were not systematically analyzed. Future studies should therefore include multi-reader assessments and blinded evaluations to better assess reproducibility and workflow efficiency.

## Conclusion

In conclusion, our study suggests that ascites volume exerts a threshold-dependent prognostic effect in HCC patients treated with Atezolizumab/Bevacizumab. While ascites volume as a continuous variable was not independently prognostic, stratification according to ascites burden identified high-volume ascites as an independent marker of poor outcome. These findings support the concept that ascites should be staged quantitatively in stages rather than treated as a binary parameter and highlight the need for further studies to refine volumetric ascites stages in systemic therapy for HCC.

## Data Availability

The datasets generated and analysed during the current study are not publicly available due to them containing information that could compromise patient privacy. However, data are available from the corresponding author on reasonable request.

## Abbreviations

AASLD: American Association for the Study of Liver Diseases
AI: Artificial Intelligence
AIH: Autoimmune Hepatitis
ALBI: Albumin-Bilirubin (Score)
BCLC: Barcelona Clinic Liver Cancer
CI: Confidence Intervals
CT: Computed Tomography
EASL: European Association for the Study of the Liver
HBV: Hepatitis B Virus
HCC: Hepatocellular Carcinoma
HCV: Hepatitis C Virus
HR: Hazard Ratios
MASH: Metabolic Dysfunction-Associated Steatohepatitis
OS: Overall Survival
PACS: Picture Archiving and Communication System
PBC: Primary Biliary Cholangitis
TACE: Transarterial Chemoembolization
